# Developing a Japanese Version of the Baron Depression Screener for Athletes among Male Professional Rugby Players

**DOI:** 10.3390/ijerph17155533

**Published:** 2020-07-31

**Authors:** Yasutaka Ojio, Asami Matsunaga, Kensuke Hatakeyama, Shin Kawamura, Masanori Horiguchi, David Baron, Chiyo Fujii

**Affiliations:** 1 Department of Community Mental Health & Law, National Institute of Mental Health, National Center of Neurology and Psychiatry, Tokyo 187-8553, Japan; 2 Japan Rugby Players Association, Tokyo 108-0074, Japan; 3 Senior Vice President and Provost, Western University of Health Sciences, Pomona, CA 91766, USA

**Keywords:** athlete, mental health, depression, anxiety, screening

## Abstract

The Baron Depression Screener for Athletes (BDSA) is a brief, valid, and reliable athlete- specific assessment tool developed in the US to assess depressive symptoms in elite athletes. We examined the applicability and reliability of a Japanese version of the BDSA (BDSA-J) in a Japanese context, and further examined the construct validity of the BDSA-J. Web-based anonymous self-report data of 235 currently competing Japanese professional male rugby players (25–29 years = 123 [52.3%]) was analyzed. A two-stage process was conducted to validate the factor structure of the BDSA-J using exploratory factor analysis in a randomly partitioned calibration sample, and confirmatory factor analysis in a separate validation sample. Cronbach’s alpha was used to assess internal consistency. Spearman’s rank-order correlation coefficients were calculated to examine convergent validity with the Kessler-6. We identified a one-factor structure for BDSA-J. Confirmatory factor analysis supported this one-factor model, revealing good model fit indices. The standardized path coefficients for each of the items were β = 0.52 to 0.79 (*p* < 0.001). A Cronbach’s alpha of 0.71 was obtained for the BDSA-J. BDSA-J showed significant positive correlations with the Kessler-6. The BDSA-J is an appropriate and psychometrically robust measure for identifying depressive symptoms in Japanese male rugby players.

## 1. Introduction

Expert opinions and statements of several international sports committees have been published over the past few years, and mental health support for elite athletes has attracted rising attention [1,2,3,4,5,6,7]. These academic and practical movements are based on findings from epidemiological surveys [8,9,10,11,12,13,14,15,16,17], mainly in the United States, European countries, and Australia. The statements focus on the importance of early detection and appropriate support for mental health problems. Therefore, mental health screening is an essential process in a comprehensive framework for athlete mental health support strategies [18,19].

The statements have been based on a number of surveys about the mental health of professional athletes, but have not included any data or survey of Japanese elite athletes, except for one study targeted at junior athletes [20]. One reason for this is the lack of a mental health assessment tool for Japanese elite athletes. This issue may also lead to a delay in the understanding of the mental health status of Japanese athletes. In Japan, as in other countries, based on the mental health framework for athletes indicated in the statements, the development of a mental health assessment tool is required as an element necessary to realize appropriate mental health support for elite athletes.

The International Olympic Committee’s Expert Consensus Statement on mental health in elite athletes has recommended utilizing the Baron Depression Screener for Athletes (BDSA) [6,21]. The BDSA is a brief depression screening tool specifically for use with athletes. Such an athlete-specific measure of mental health problems is necessary to advance our understanding and more effectively support athletes’ mental health and performance [21]. To the best of our knowledge, little is known about the applicability of this BDSA to Japanese elite athletes. The development of the Japanese version of BDSA (BDSA-J) may contribute to improving the mental health promotion and support for Japanese elite athletes. Therefore, this study aims to develop and validate a Japanese version of the BDSA in a sample of Japanese male professional rugby players, that was feasible, based on the available data.

## 2. Materials and Methods

### 2.1. Participants

The participants were a total of 600 male, professional athletes from the Japan Rugby Players Association who provided data. All the athletes were aged 18 years and over. No exclusion criteria were applied.

### 2.2. Procedure

This study was approved by the Research Ethics Committee at the National Center of Neurology and Psychiatry, Japan (NCNP) (approval number: A2020-015). The questionnaires were distributed to all athletes who enrolled in the Japan Rugby Players Association. Questionnaires were completed only by athletes who voluntarily consented to participate in the study. All the participants were invited to complete an anonymous online survey, which took approximately 10 min to complete. The participants were informed of the aim of the study, data collection procedures, and the implications of participation or non-participation in this study via the cover page of the questionnaire on the web. The participants were reminded of any missing items prior to progressing to the next page, resulting in no missing outcome data. The participants were provided with individual access to a tablet or laptop computer to complete the survey.

### 2.3. Measurements

#### 2.3.1. Baron Depression Screener for Athletes

The BDSA is a 10-question self-report questionnaire that addresses mood, sports-related anhedonia, weight loss, fatigue, self-image, substance abuse, suicidality, and other parameters over the previous 2 weeks (Table S1), and has been validated against the Beck Depressive Inventory [21,22]. According to Dr. Baron [21], the developer of this scale, the scale has no cut-off scores that automatically suggest a diagnosis of major depressive disorder. The scale consists of 10 items on a three-point Likert scale (range 0 to 20, a higher score representing more severe depressive symptoms). The following steps were conducted in order to translate the BDSA into Japanese. The original BDSA was translated into Japanese by the first author (Y.O.). The professional athletes (K.H. and S.K.) in this research team modified the terms and sentences in order to improve the readability for athletes. A back-translation was produced by a bilingual English speaker. Finally, the back-translated version of the BDSA was confirmed and approved by the researcher who had originally developed the BDSA (D.B.).

#### 2.3.2. Other Measurements

To assess convergent validity of the BDSA-J in the current study, we used the Kessler-6 (K6), which is one of the most widely used measures of global psychological distress [23]. Performance of the K6 in detecting mood and anxiety disorders, as assessed by the areas under the receiver operating characteristic curves (AUCs), was excellent, with values as high as 0.86, 0.89, and 0.94 in the US [24], Australian [25], and Japanese general sample [26], respectively. In the K-6, the scores are categorized to indicate the respondents’ mental health problems over the previous 30 days. Responses to items are made on a 5-point scale. The K-6 was developed and validated based on many epidemiological surveys and is widely used as a useful screening tool in assessing treatment progress in common mental disorders such as anxiety and depression in people in general. The K-6 has demonstrated robust psychometric properties in Japanese populations [26].

### 2.4. Validity and Statistical Methods

A two-stage process was undertaken to validate the factor structure of the BDSA-J using exploratory factor analysis (EFA) in a randomly partitioned calibration sample, and confirmatory factor analysis (CFA) in a separate validation sample. First, exploratory factor analysis was undertaken to determine the underlying factor structure of the BDSA-J. To determine which items belonged to each factor, we extracted items if they loaded ≥0.3. In addition, the number of factors was determined based on scree plots and parallel analysis. For estimation in both EFA and CFA, the responses for each item were assumed to be ordinal variables, and a weighted least square mean and variance (WLSMV) adjusted estimator was used due to the highly skewed distribution and 0–2 range of the BDSA-J score. In addition, for the CFA, because of the skewed distribution and narrow range of BDSA-J score of each item, the parceling method from research by Matsunaga [27] was adopted to alleviate skewness and expand the score range of BDSA-J used in CFA. Random number generation was performed for the ten items of the BDSA-J, and parcels were generated which were identified by random numbers. Since the parcels were generated by aggregating item scores and the BDSA item score ranged from 0–2, each of the item scores of individuals was transformed by adding one point to retain the intervals of the Likert scale after the parceling. The paper by Matsunaga (2008) [27] recommended generating more than three parcels for each factor. We generated a total of five parcels with two items for the one-factor structure of BDSA to divide equally all ten items. We adopted the typically accepted criteria to assess the model fit, the comparative fit index (CFI) > 0.95, the Tucker– Lewis fit index (TLI) > 0.95, root mean square error of approximation (RMSEA) < 0.50. After confirming the factor structure, we examined the internal consistency and convergent validity. To evaluate internal consistency, Cronbach’s alpha was calculated. In terms of convergent validity, Spearman’s rank-order correlation coefficients were calculated to determine if the BDSA-J had a positive correlation with the K-6. All analyses were conducted with Stata version 16 (StataCorp LLC, College Station, TX, USA) and Mplus 8.2 software (Muthén & Muthén, Los Angels, CA, USA).

## 3. Results

We obtained consent from 235 participations (response rate: 39.1%). Table 1 shows the demographic characteristics of the participants in this study. Over half of the participants (52.3%) were 25–29 years old, with between 30 and 34 years (24.7%) or 20 and 24 years (19.6%), or over 35 years (3.0%) or under 19 (0.4%). A total of 97.5% had graduate university educational attainment. A total of 48.1% of them were married and 28.9% had a child in the family. About half of them lived with family or a partner. A total of 19.2% of them had experience in the national team, and 37.9% reported that they did not play in a competition last season.

**Figure 1:**
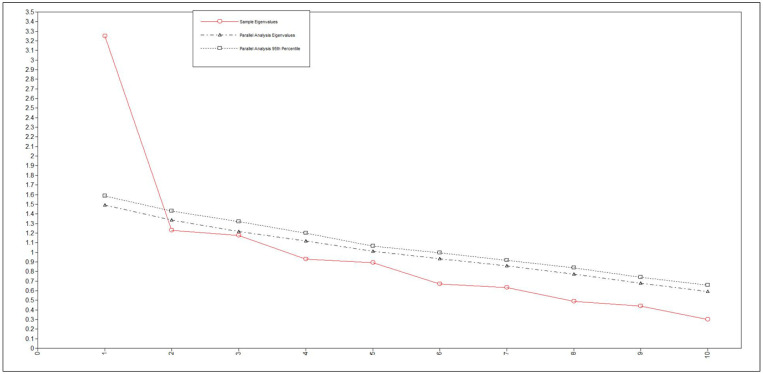
Scree plots and parallel analysis for BDSA-J.

The distribution of participants’ responses and factor loadings from the EFA and the means and SD for the BDSA-J are shown in Table 2. Considering the results of the scree plots and parallel analysis, we employed a one-factor structure. When the one-factor structure was employed, the factor loading values for all the items were ≥0.3. Scree plots for BDSA-J are shown in Figure 1.

**Figure 2:**
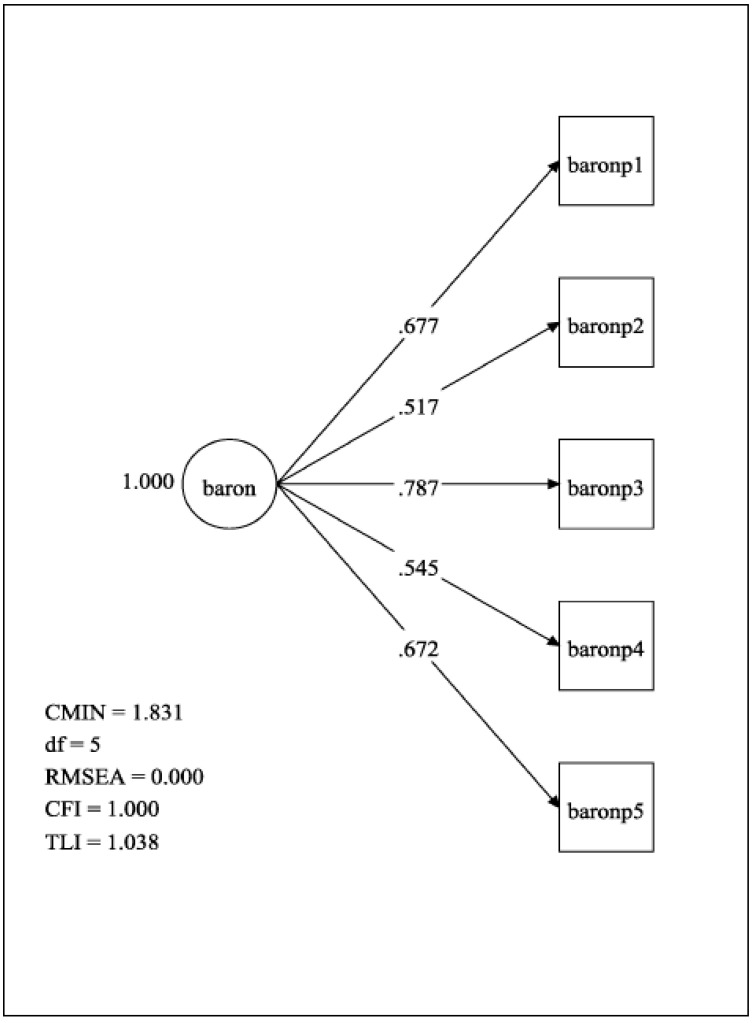
Confirmatory factor analysis of 10-item BDSA-J with one-factor structure. Abbreviation: Chi-square statistics (CMIN); root mean square error of approximation (RMSEA); comparative fit index (CFI); Tacker-Lewis fit index (TLI).

The results of CFA are presented in Figure 2. The one-factor model of BDSA-J showed the following model fit indices: Chi-square statistics = 1.831, df = 5; RMSEA = 0.000; CFI = 1.000; TLI = 1.038. The standardized path coefficients for each item were β = 0.52 to 0.79 (*p* < 0.001).

The Item-test, Item-rest correlation for the 10-items BDSA-J is shown in Table 3. The Cronbach’s alpha (α) of 0.71 was obtained for the BDSA-J. BDSA-J showed significant and positive correlations with the K-6 (ρ = 0.489, *p* < 0.001). These indicate acceptable internal consistency and good convergent validity of the BDSA-J.

## 4. Discussion

We developed and evaluated the psychometric properties of the Japanese version of BDSA. The current findings from Japanese male professional rugby players confirm the validity, reliability, and factor structure. In factor analyses, BDSA-J demonstrated a one-factor structure the same as the original version of BDSA. The BDSA-J showed good psychometric properties, with relatively high Cronbach’s alphas, as well as convergent validity with the K-6. The one-factor screening tool, the BDSA-J may be a useful assessment tool for examining mental health problems in male rugby players, especially with symptoms of depression.

Our findings suggest that the BDSA-J ensures good convergent validity with the K-6. The advantage of using BDSA-J in elite athlete populations may be that BDSA-J items also provide important information relating to athletes’ mental health support. The BDSA-J has more items than the K-6, but still has only 10 items, which makes it a sufficiently brief assessment tool. In addition, BDSA-J has the great advantage of being able to assess externalization problems related to mental health, including alcohol and substance abuse, and the presence or absence of suicidal ideation. In a population that tends to be predominantly male, such as elite athletes, not only internalization problems but externalization problems are often observed as symptoms of depression [28,29]. BDSA- J, which can assess both internalization and externalization issues, might be a useful tool for assessing depressive symptoms in elite athletes. Focusing on the response to each item may be helpful in providing practical mental health support for athletes. For the translation in the development process of BDSA-J, we adjusted athlete-friendly terms together with active elite athletes. The co-production with elite athletes may have improved the accuracy of responses to the questions of BDSA-J [30].

The current result is obtained through the survey for professional rugby players. Rugby is a team sport as a protective factor for mental health problems [31], albeit being a contact sport is an evident risk factor [32]. In particular contact sports, such as rugby are related to chronic traumatic encephalopathy (CTE). Previous papers have suggested that the symptom of CTE present as a form of dementia with characteristics approachable to front-temporal dementia in the later age of retired athletes [33], even in athletes of younger age, the mental health-related behavioural symptoms including depression alongside cognitive impairment and anger control problems may commence insidiously as CTE signs [34]. In addition, other research has pointed out that CTE, mood disorder, and suicidality have mutual and complex associations with each other [35,36]. Therefore, the possibility that some symptoms detected by the BDSA-J may suggest CTE and its related symptoms cannot be rigidly excluded. Considering this aspect, we might allege the possibility of its prevention including wearing helmets in contact sports.

The current results indicate that the BDSA-J might help early detection of mental health problems in elite athletes and may enable effective early intervention in mental illness. The implementation of the BDSA-J in combination with an existing support program over an athlete’s career might be an effective strategy. The player development program (PDP) that provides holistic support with well-being and mental health including referral to experts has been widely adopted among rugby societies in Australia and New Zealand [37,38]. In addition, the Australian Institute of Sports (AIS) recently established a national Mental Health Referral Network that employs a decentralized model across Australia [39]. In particular, it would be useful if BDSA-J could be used during periods associated with a high risk of rising mental health problems in athletes, such as injuries and/or retirement [12,32,40]. However, it should be noted that the results of this study did not reveal a long-term predictive association between BDSA-J scores and their high-risk factors. Prospective studies are needed to establish the long-term predictive effects of BDSA-J on the performance and health of elite athletes. According to a previous paper [41], sharing the protocol of mental health-related project with stakeholders, such as service providers and users, may increase their mental health knowledge, which can create more specific health promotion and prevention programs. Our research team launched a research project to create mental health promotion and care systems for professional athletes in Japan through the qualitative and quantitative mixed methods approach as Co-production with the Japan Rugby Players Association [42].

### Strength and Limitations of this Rearch

We recognize that the great strength of this study is that it is the first mental health survey for Japanese professional athletes, to develop a psychometrically robust measurement for identifying mental health problems. We also recognized several limitations that need to be considered with our findings. First, the current samples were all male professional rugby players. We used the male-only sample because it was feasible based on the data available in this survey. The 39.1% response of this survey is not lower than other mental health surveys [43], but the rate might also have been affected by mental health-related stigma toward mental health problems [44]. To better generalize the findings of this study, future research should evaluate the psychometric properties of BDSA-J in other sports and with female elite athletes. Second, the test–retest stability of BDSA-J could not be evaluated because of the cross-survey of the current study. We need to address this and ideally investigate trajectories over time. Third, the factors that affect BDSA-J scores should be considered. There should be a focus on the effectiveness of scale convergence and divergence, including assessments of critical stages of risk, such as the duration of injury and the transition from competitive sports. Future studies should address these challenges to enhance the evidence on BDSA-J.

## 5. Conclusions

In the field of mental health for elite athletes, clinical and research interests are growing internationally, and there is a requirement to develop mental health services for elite athletes. In order to promote the development of such mental health services and their social implementation, there was a great need for a brief and effective assessment tool specifically for elite athletes. BDSA-J enables the assessment of mental health problems, including depressive symptoms, in elite athletes. By utilizing this scale, we may be able to support athletes in their mental health, including early detection and early intervention in psychiatry.

## Figures and Tables

**Table 1:** Demographic characteristics of the study participants.

	% (*n*)
Age at survey	
~19	0.4 (1)
20–24	19.6 (46)
25–29	52.3 (123)
30–34	24.7 (58)
35~	3.0 (7)
Educational attainment	
High school	0.85 (2)
Four-year college or university	97.5 (229)
Postgraduate college (or higher)	1.7 (4)
Marital status	
Married	48.1(113)
Never married	51.1 (120)
Divorced or widowed	0.9 (2)
Child living in household	
No	71.1 (167)
Yes	28.9 (68)
Residential Status	
Living alone	17.5 (41)
Living with family and/or partner	50.6 (119)
Dormitory	31.9 (75)
Experience of national team	
No	80.9 (190)
Yes	19.2 (45)
Playing status of last season	
As a active member	29.8 (70)
As a reserve member	32.3 (76)
No play	37.9 (89)

**Table 2:** Factor loadings for BDSA-J items.

Item No.	Statement	Factor Loadings
1	I feel sad even after a good practice session or successful competition.	0.59
2	I rarely get pleasure from competing anymore and have lost interest in my sport.	0.70
3	I get little or no pleasure from my athletic successes.	0.35
4	I am having problems with my appetite and weight.	0.46
5	I do not feel rested and refreshed when I wake up.	0.60
6	I am having problems maintaining my focus and concentration during training and competition.	0.70
7	I feel like a failure as an athlete and person.	0.78
8	I cannot stop thinking about being a failure and quitting sports.	0.84
9	I am drinking alcohol or taking supplements to improve my mood.	0.37
10	I have thoughts of ending my life.	0.71

**Table 3:** Item-test, item-rest correlation and Cronbach’s alpha of the BDSA-J.

Item No.	Item-Test Correlation	Item-Rest Correlation	α
1	0.53	0.40	0.68
2	0.61	0.49	0.67
3	0.39	0.15	0.74
4	0.47	0.26	0.71
5	0.60	0.46	0.67
6	0.65	0.52	0.66
7	0.65	0.51	0.66
8	0.69	0.55	0.65
9	0.42	0.25	0.71
10	0.29	0.20	0.71
Test scale			0.71

## Supplementary Materials

The following are available online at https://www.mdpi.com/1660-4601/17/15/5533/s1, Table S1: Proportion of participants’ responses to the Japanese version of the Baron Depression Screener for Athletes (BDSA-J) (*n* = 235).
