# Supplementary material for: Developing a Japanese Version of the Baron Depression Screener for Athletes among Male Professional Rugby Players
Source: Int J Environ Res Public Health. 2020 Jul 31;17(15):5533. doi: 10.3390/ijerph17155533 (PMC7432524; doi:10.3390/ijerph17155533)
Supplement: Supplementary file 1 [file ijerph-17-05533-s001.pdf]

Supplementary Materials

Table S1. Proportion of participants' responses to the Japanese version of the Baron Depression Screener for Athletes (BDSA-J) (n = 235).

Item no.	Statement	Never % (n)	Some of the time (over a 2-week period) % (n)	Most of the time (over a 2-week period) % (n)	Mean (SD)
1	I feel sad even after a good practice session or successful competition.	76.6 (180)	21.7 (51)	1.7 (4)	0.25 (0.47)
2	I rarely get pleasure from competing anymore and have lost interest in my sport.	64.7 (152)	33.6 (79)	1.7 (4)	0.37 (0.52)
3	I get little or no pleasure from my athletic successes.	52.3 (123)	30.6 (72)	17.0 (40)	0.65 (0.76)
4	I am having problems with my appetite and weight.	60.0 (141)	27.7 (65)	12.3 (29)	0.52 (0.71)
5	I do not feel rested and refreshed when I wake up.	17.9 (42)	70.2 (165)	11.9 (28)	0.94 (0.54)
6	I am having problems maintaining my focus and concentration during training and competition.	40.9 (96)	53.6 (126)	5.5 (13)	0.65 (0.58)
7	I feel like a failure as an athlete and person.	56.6 (133)	38.7 (91)	4.7 (11)	0.48 (0.59)
8	I cannot stop thinking about being a failure and quitting sports.	45.5 (107)	46.4 (109)	8.1 (19)	0.63 (0.63)
9	I am drinking alcohol or taking supplements to improve my mood.	60.0 (141)	35.7 (84)	4.3 (10)	0.44 (0.58)
10	I have thoughts of ending my life.	91.9 (216)	8.1 (19)	0 (0)	0.08 (0.27)

Calculation of mean score: Never = 0, Some of the time = 1, Most of the time = 2
